# It is Separation, Not Contact: Electrification at Water–Hydrophobe Interfaces during Wetting–Dewetting Cycles

**DOI:** 10.1021/acs.langmuir.5c05487

**Published:** 2026-01-30

**Authors:** Yinfeng Xu, Himanshu Mishra

**Affiliations:** † Environmental Science and Engineering (EnSE) Program, Biological and Environmental Science and Engineering (BESE) Division, 127355 King Abdullah University of Science and Technology (KAUST), Thuwal 23955-6900, Saudi Arabia; ‡ Sustainable Food Security Center of Excellence, King Abdullah University of Science and Technology (KAUST), Thuwal 23955-6900, Saudi Arabia; § Interfacial Lab (iLab), King Abdullah University of Science and Technology (KAUST), Thuwal 23955-6900, Saudi Arabia

## Abstract

When water contacts hydrophobic materials—such as air, hydrocarbons, or fluorocarbons–the interface acquires charge, yet how dynamic wetting–dewetting governs this electrification remains largely unexplored. Here, using controlled pipetting experiments with hydrophobic capillaries, we show that electrification at water–hydrophobe interfaces is governed by liquid–solid separation rather than contact formation. By systematically varying liquid uptake and release rates over 3 orders of magnitude, we find that the charge transferred during a pipetting cycle depends nonlinearly on the velocity and acceleration of the receding liquid meniscus, while the advancing (uptake) motion contributes negligibly. High-resolution charge measurements reveal that, although net charge is conserved, the charge generated during liquid release in a given cycle directly influences the charge acquired during liquid uptake in the subsequent cycle. These observations uncover a previously unrecognized intercycle coupling in water–hydrophobe electrification and demonstrate that charge conservation is more appropriately described across successive wetting–dewetting cycles rather than strictly within an individual cycle. This intercycle formulation accurately captures charge balance under dynamically varying flow conditions and resolves apparent inconsistencies observed when release rates are changed between cycles. These findings hold across hydrophobic capillaries with negative, near neutral, and positive surface charge densities. Thus, our report establishes liquid–solid separation kinetics as the dominant control parameter for electrification at water–hydrophobe interfaces and highlight the inherently history-dependent nature of interfacial charging. These insights advance the fundamental understanding of water–hydrophobe electrification and have implications for droplet-based technologies, micro- and nanofluidics, and liquid-handling processes.

## Graphical abstract

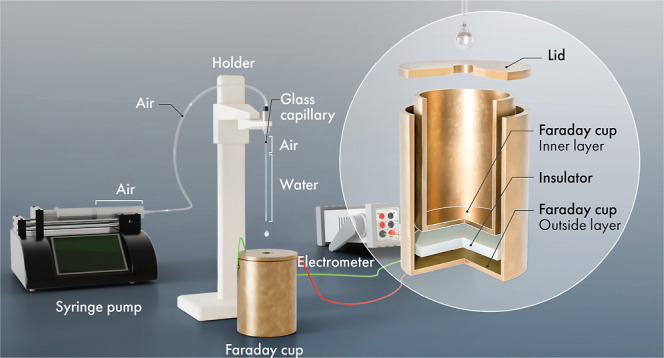

## Introduction

Water interfaces with hydrophobic materials (“hydrophobes”, spanning solid, liquid, and gas phase) are ubiquitous in nature and technology, underpinning phenomena atmospheric and aerosol chemistry to agriculture, separation processes, and micro- and nanofluidics.^1–16^ A defining and widely reported feature of these interfaces is their tendency to acquire negative electric charge when water comes into contact with a hydrophobe. This induces a positive charge in the bulk water adjacent to the interface, i.e., the electrical double layer, and has been suggested to influence the physical and chemical response of interfacial systems.^17–19^ For example, when water is drawn into a hydrophobic capillary and subsequently expelled as a pendant droplet, it carries a net positive charge.^20,21^ This observation is often linked to the mobility of bubbles and oil droplets under electric fields, leading to the generalization that water–hydrophobe interfaces are negatively charged.^22–26^ Despite considerable experimental, theoretical, and computational effort to unravel the nature of this negative charge, a clear consensus has not yet emerged.

In the literature, several–often contradictory and intensely debated–mechanisms have been reported. These include the interfacial adsorption of the intrinsic ions of water (i.e., hydroxide or protons),^27–33^ deprotonation of surface groups,^34,35^ enhanced autolysis of water at hydrophobic interfaces,^25^ partial charge transfer between water molecules^36^ and between water and hydrophobes,^37^ dipolar organization of interfacial water,^38,39^ airborne surfactants or impurities,^40–42^ adsorption of bicarbonate ions arising from ambient carbon dioxide,^43^ volta potentials,^44^ and cryptoelectrons.^45^

These mechanisms have been interrogated using a wide range of experimental approaches, including surface-specific spectroscopic measurements–typically employing concentrated salty, acidic, or basic solutions^46^–direct surface force measurements,^34,47,48^ and direct measurements of droplet charge using electrometers or droplet deflection inside uniform electric fields.^20,44,49,50^ Complementary experiments involving impinging or sliding droplets on dielectrics (typically hydrophobic) surfaces with underlying electrodes have laid the foundation for droplet-based electricity generators and clarified the effects of the surface chemistry, mechanical stiffness, and flow discontinuities.^51–62^ Notably, Wong et al.^63^ demonstrated charge reversal at water–hydrophobe interfaces via sliding droplet experiments. However, such experiments are generally confined to a narrow range of droplet velocities (≈0.1–0.5 m·s^–1^), while droplet spreading and retraction during impact generate contact-line velocities spanning roughly 0.3–3 m·s^–1^.^51–63^ Taken together, these observations underscore the need for a systematic assessment of how the rate of liquid–solid contact formation and, critically, separation governs electrification at water–hydrophobe interfaces.

In a classic experiment, Faubel & Steiner showed that electrification during high-speed liquid motion depends on the velocity of water flowing along solid surfaces.^64^ Building on this observation, Saykally & co-workers^65^ demonstrated hydrogen production and electrical power generation from electrokinetic flows merging from hydrophobic capillaries at velocities of 50–400 m·s^–1^. Within a modified Poisson–Boltzmann framework combined with continuum hydrodynamics, these studies predict streaming currents that increase linearly with flow velocity.^65,66^ However, these predictions appear to contrast sharply with more recent studies of dewetting-induced charge separation. Ratschow and co-workers investigated sliding droplets on hydrophobic surfaces and reported a decrease in charge separation with increasing droplet velocity, attributing this behavior to velocity-dependent expansion of the diffuse electrical double layer at the receding contact line.^67^

Taken together, these studies highlight differing dependencies of electrification on liquid velocity across experimental regimes, ranging from steady, high-speed electrokinetic flows to transient droplet motion. However, most existing experiments probe either steady-state flows or sliding droplets within limited velocity ranges, leaving comparatively unexplored the transient, finite-volume regime characteristic of cyclic liquid uptake and release. This gap motivates a systematic examination of how the kinetics of liquid–solid contact formation and separation influence electrification during wetting–dewetting cycles.

In this work, we address these gaps using a controlled pipetting geometry, in which water is repeatedly drawn into and released from hydrophobic capillaries to form pendant droplets. This simple yet well-defined configuration allows us to decouple liquid–solid contact formation from separation and to quantify charge transfer during each stage of a wetting–dewetting cycle. Specifically, we ask.

(i) how does electrification during a single pipetting cycle depend on the velocity and acceleration of the liquid as it enters or leaves the capillary
(ii) do liquid uptake and release contribute symmetrically to charge transfer; and
(iii) how should charge conservation be formulated during sequential wetting–dewetting cycles under dynamically varying flow conditions?

Our results reveal that electrification is governed primarily by the kinetics of liquid release, while liquid uptake plays a negligible role. Moreover, we find that charge generated during liquid release in one cycle directly influences charge acquisition during uptake in the subsequent cycle, necessitating an intercycle formulation of charge conservation.

## Results and Discussion

### Surface Characterization and Charge Reversal

To investigate electrification at water–hydrophobe interfaces, we employed borosilicate glass capillaries whose surfaces were chemically modified to produce hydrophobic interfaces with tunable surface charge. Glass capillaries were functionalized using octadecyltrichlorosilane (ODTS) to generate negatively charged water–hydrophobe interfaces, following previously reported protocols.^9,68^ Briefly, the surface was activated via a piranha treatment, followed by silanization with octadecyltrichlorosilane (ODTS) in a toluene solution. The water–ODTS interface, similar to common water–hydrocarbon interfaces, has a negative surface charge density.^21,59^ To broaden the study to interfaces with near-neutral and positive charge, we adopted the approach introduced by Wong et al.,^63^ in which activated glass surfaces were briefly exposed to (3-aminopropyl)­triethoxysilane (APTES) prior to ODTS functionalization. By controlling the APTES exposure time, we obtained three surface treatments: ODTS, APTES (2 s)–ODTS), and APTES (5 s)–ODTS. Advancing and receding contact angle measurements confirm that all surfaces exhibited hydrophobicity (Supporting Information Note S1).

We first quantified the baseline electrification of these capillaries with the uptake and release rates set at 10 mL·min^–1^ using a syringe pump. A fixed volume of water (50 μL, Milli-Q, 18.2 MΩ cm) was withdrawn from an electroneutral reservoir and dispensed as a pendant droplet into a Faraday cup connected to an ultrasensitive electrometer with a limit of detection of 0.1 pC (Figure 1a). Upon droplet release, the measured charge increased sharply and stabilized within ∼2 s (Figure 1b). Measurements were repeated at least ten times for each surface treatment, and the mean charges are reported with ±1 standard deviation (Figure 1c).

**Figure 1:**
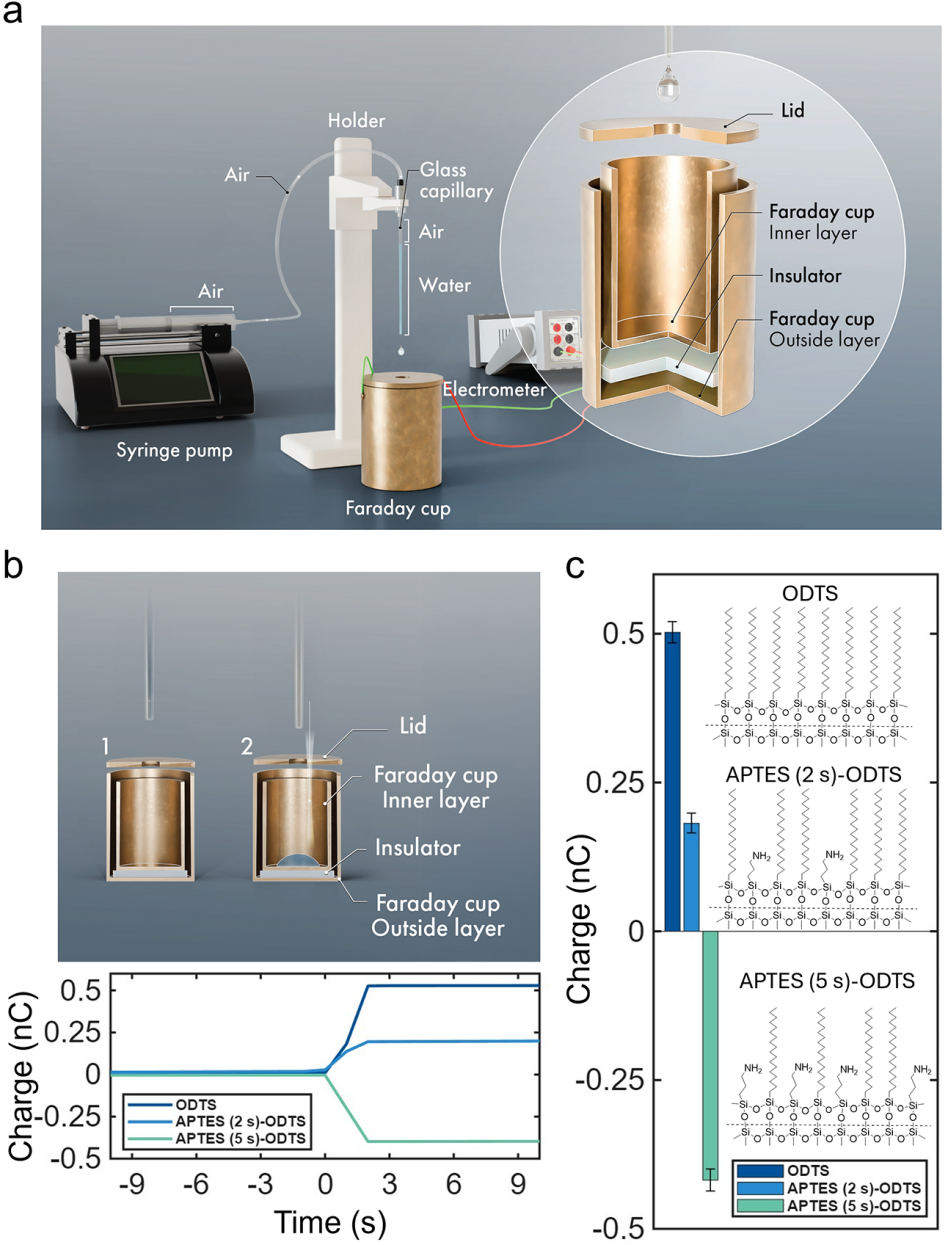
Baseline electrification of water droplets dispensed from hydrophobic capillaries of controlled chemical makeup. (a) Experimental setup consisting of a copper Faraday cup connected to an ultrasensitive electrometer (detection limit: 0.1 pC). (b) In a typical experiment, water is withdrawn from an electroneutral reservoir using a capillary and is dispensed as a single droplet into the Faraday cup (both rates: 10 mL·min^–1^). When the droplet lands in the Faraday cup, the resulting charge is recorded. Representative single measurements for all three surface treatments are presented. The time origin (*t* = 0) corresponds to the moment at which the droplet is released. (c) Mean charge carried by droplets dispensed at a rate of 10 mL min^–1^ from capillaries coated with ODTS, APTES (2 s)–ODTS, and APTES (5 s)–ODTS, demonstrating tunable charge polarity via surface chemistry. Error bars represent ±1 standard deviation. ODTS and APTES denote octadecyltrichlorosilane and (3-aminopropyl)­triethoxysilane, respectively.

At a dispensing rate of 10 mL min^–1^, droplets released from ODTS-coated capillaries carried a net positive charge of +0.50 ± 0.03 nC. Assuming charge neutrality of the initial reservoir, this value corresponds to a negative surface charge density on the capillary wall of −3.1 × 10^–6^ C m^–2^, in reasonable agreement with literature values obtained using similar methods.^20,21^ In contrast, droplets released from APTES (2 s)–ODTS and APTES (5 s)–ODTS capillaries carried net charges of +0.18 ± 0.02 nC and −0.42 ± 0.02 nC, respectively, demonstrating tunable charge polarity via surface chemistry (see Supporting Information Note S1 for mechanistic insights).

These baseline measurements establish two key points. First, the sign of electrification is governed by the surface chemistry of the hydrophobe rather than by the water itself. Second, the capillary–droplet system provides a reproducible platform for quantifying interfacial charge transfer, enabling systematic investigation of how flow conditions influence electrification, which we examine next.

### Flow-Rate Dependence of Electrification

Next, we evaluated the effects of the rates of water uptake and release (50 μL) on the charge carried by the released droplets. To gain deeper insight, we decomposed each water uptake/release cycle from a water reservoir placed inside the Faraday cup into four stages (Figure 2a) and quantified the corresponding charges: (i) contact (*Q*_1_) as a dry capillary enters the water reservoir; (ii) liquid uptake (*Q*_2_) as water is uptaken into the capillary; (iii) capillary lift (*Q*_3_), where the filled capillary is removed from the reservoir (and the electrometer); and (iv) liquid release (*Q*_4_), releasing the liquid back into the reservoir. *Q*_*i*_ denotes the net charge recorded by the electrometer during stage *i*(*i* = 1,2,3,4). A positive sign indicates a net gain of a positive charge at the electrometer.

**Figure 2:**
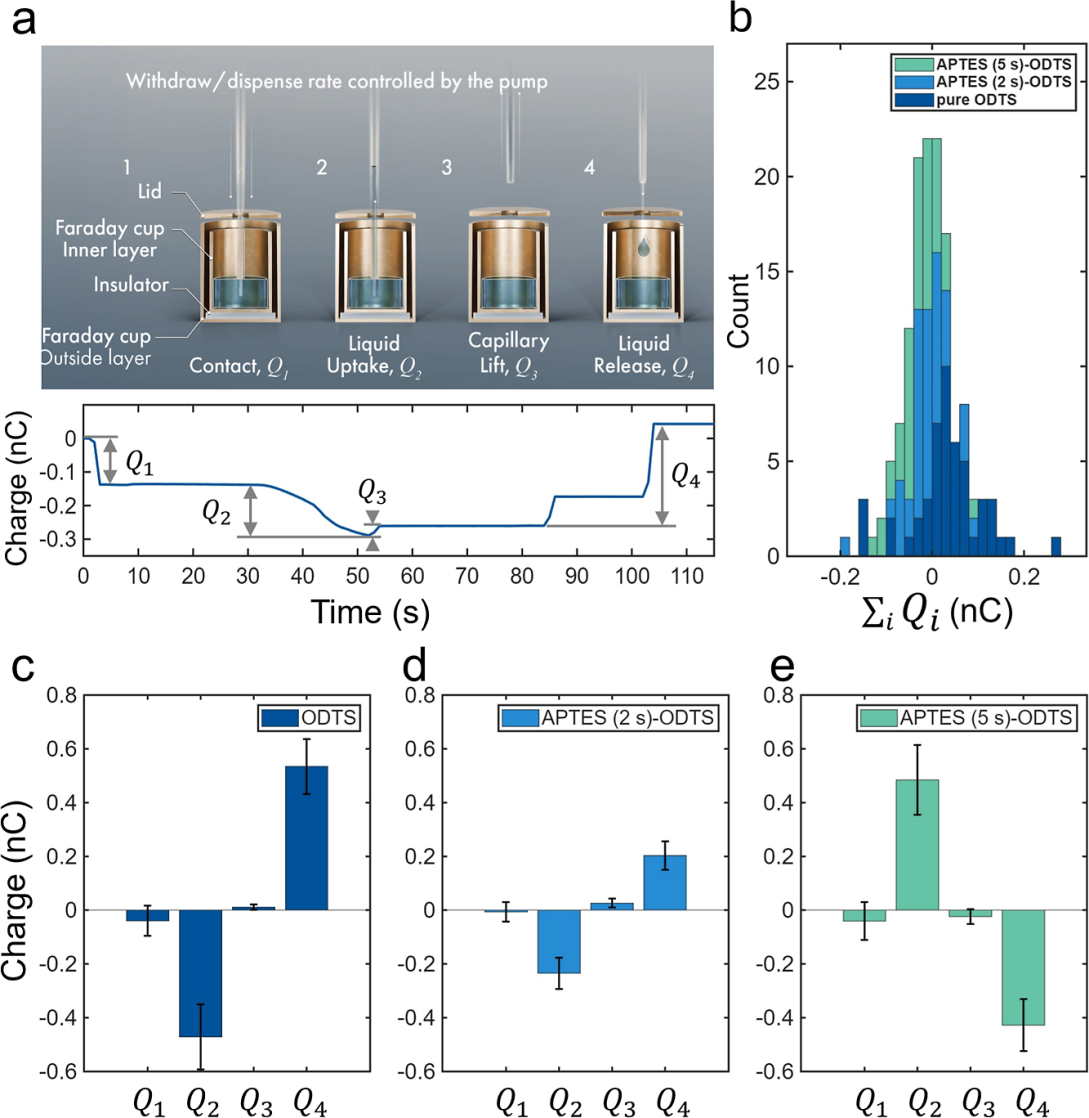
Charge measurements as a function of the uptake and release rates of 50 μL of water to and from the hydrophobic capillaries. (a) Four major stages of liquid dispensing: contact, liquid uptake, capillary lift, and liquid release, with corresponding charges. The charge curve corresponds to representative four stages of an ODTS-coated capillary with uptake and release rates set to 1 mL·min^–1^. Data are corrected for drift. (b) Charge neutrality for each cycle. The net charge $\sum_{i=1}^{4}Q_{i}$ is statistically indistinguishable from zero across all flow rates and surface chemistries. (c-e) Charge measured for four stages with all three kinds of capillaries. During water uptake and release, *Q*_2_ and*Q*_4_ are approximately equal in size but opposite in sign, while *Q*_1_ and *Q*_3_ are comparatively smaller. Error bars represent ±1 standard deviation.

As the uptake/release rates varied from 1 to 10 to 100 mL·min^–1^, so did the speeds of the waterfront advancing or receding (i.e., wetting or dewetting) within. For a given capillary, the three release rates and the three uptake rates yielded 9 combinations in total; and for each combination, 5 back-to-back cycles were recorded. The measurement procedure and drift correction are described in Supporting Informtion Note S2. It also explains that at low release rate, two separate pendant drops would form (Figure 2a-notice the step between stages 3–4, whereas at higher rates only one pendant drop was formed).

The experimental results revealed that, for all surface treatments and uptake or release rates, the sum of the charges measured during the four stages was centered at zero (Figure 2b, i.e., the total charge was conserved). Because only a small portion of the capillary (the tip) contacted the water reservoir during the contact/lift stages, the charges measured in *Q*_1_ and *Q*_3_ were typically within ±0.1 nC and significantly smaller than *Q*_2_ and *Q*_4_ (see Figure 2c–e). Observations establish that the charge transferred during the liquid uptake step (*Q*_2_) was equal in magnitude and opposite in sign to that during the liquid release step (*Q*_4_), i.e., consistent with the charge conservation. The relatively large standard deviations observed for *Q*_2_ and *Q*_4_ arise from charge variations induced by differences in the release rate, which we will discuss in detail shortly. In comparison between *Q*_1_ and *Q*_3_, the larger error bars—and even the charge inversion observed in *Q*_1_—will be addressed later in the section on charge conservation.

Against common expectation, the experiments revealed that the rate of liquid uptake had an insignificant effect on *Q*_2_ and *Q*_4_ (Figure 3d–f and Supporting Information Note S3). In contrast, the release rate, which is simply the inverse of the uptake rate, had the most significant influence on *Q*_2_ and *Q*_4_. For instance, as the dispensing flow rate varied from 1 to 100 mL·min^–1^, the measured charge from the ODTS-coated capillaries increased from 0.43 ± 0.03 nC to 0.67 ± 0.05 nC (Figure 3a). For the APTES (2 s)–ODTS capillaries, the charge increased from 0.15 ± 0.01 nC to 0.27 ± 0.02 nC (Figure 3b). For the APTES (5 s)–ODTS capillaries, the charges varied from −0.31 ± 0.02 nC to −0.55 ± 0.02 nC (Figure 3c). As the speed of the receding water meniscus increased, so did the water–hydrophobe electrification. Please note that in each plot of Figure 3, we fix one variable while analyzing all the data collected over multiple values of uptake and release flow rates and chemical makeup. Panels in Figure 3a–c show small error bars because the release rate has a direct effect on *Q*_4_. In contrast, the standard deviations are higher in panels Figure 3d–f because the plotted data for each uptake rate contains the variations in the release rates.

**Figure 3:**
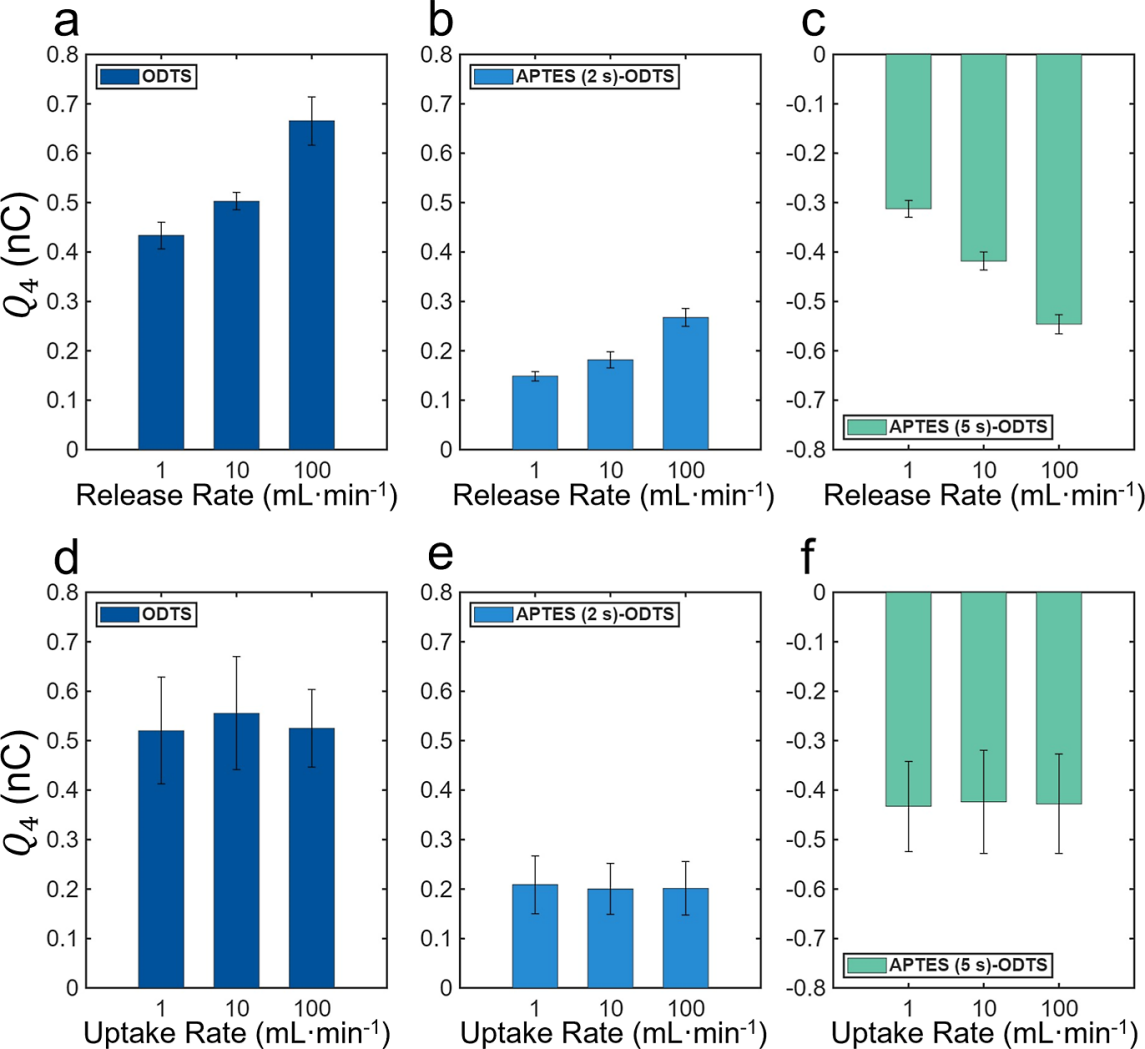
Effect of uptake/release rate to the release charge *Q*_4_ carried by 50 μL water. (a–c) Charge *Q*_4_ measured during liquid release as a function of the release rate for capillaries coated with (a) ODTS, (b) APTES (2 s)–ODTS, and (c) APTES (5 s)–ODTS, respectively, demonstrating an irrefutable effect. (d–f) As the liquid uptake rate increases, the measured *Q*_4_ reveals no significant difference for the capillaries.

The change in the liquid release rate altered both the flow speed and the duration of the water within the capillary. As the release rate critically influences electrification (Figure 3a–c), we wondered whether the lifetime of the liquid–solid contact or the speed of the liquid is the critical factor. To assess whether contact duration contributes to electrification, a controlled experiment was conducted in which the delay between uptake and release varied from 3 to 30 s, keeping the uptake and release rates constant. The net charge measured in each case exhibited no statistically significant difference, indicating that the variation in charging is not attributable to the contact time (Figure 4a).

**Figure 4:**
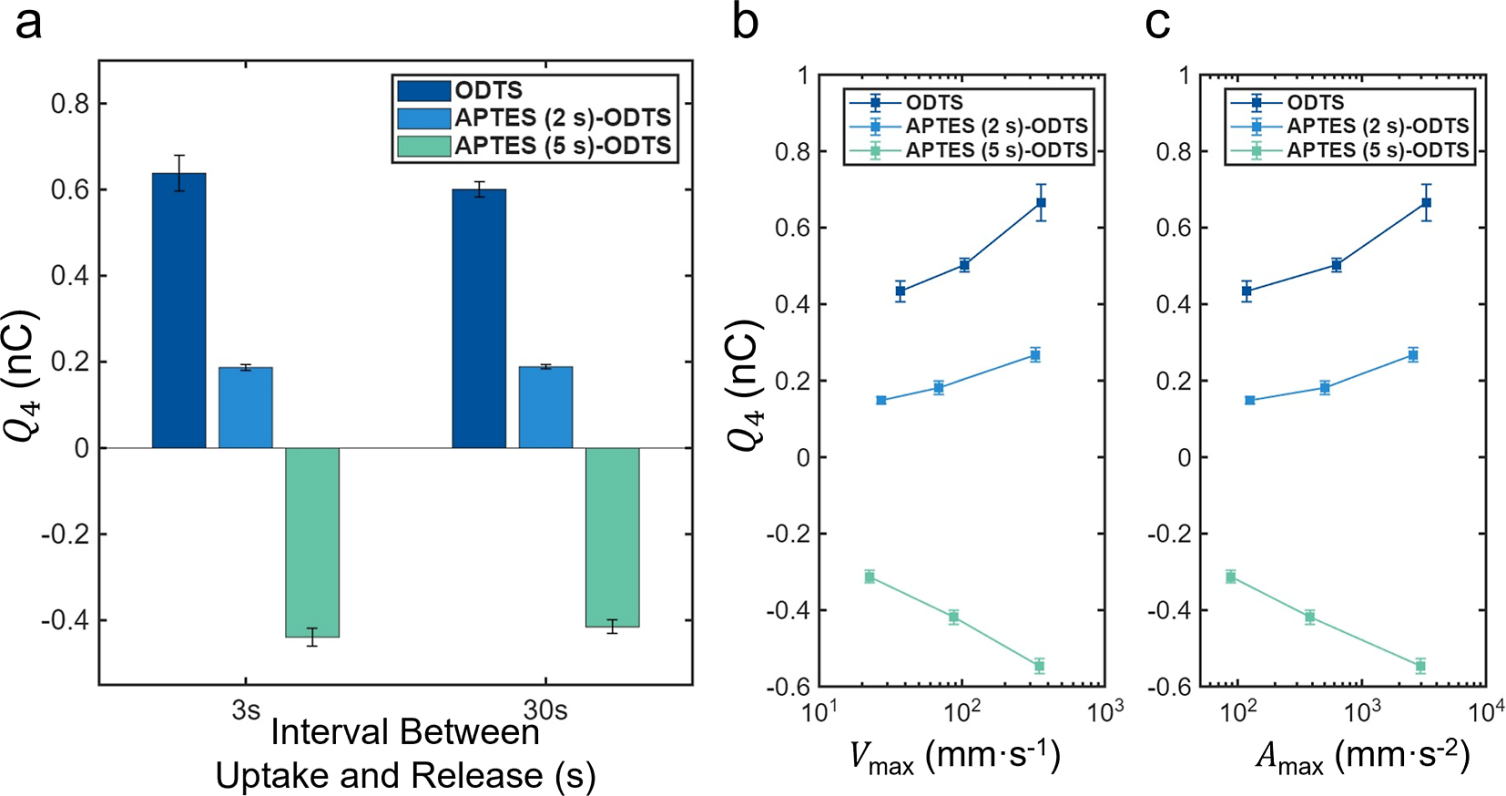
(a) Charges measured during liquid release (*Q*_4_) as a function of the time interval between the liquid uptake and release steps (3 and 30 s). (b) *Q*_4_ as a semilogarithmic function of the maximum velocity during liquid release. (c) *Q*_4_ as a semilogarithmic function of the maximum acceleration during liquid release.

Next, we quantified the speed and acceleration of the receding liquid meniscus at different release rates. High-speed imaging was used to measure the velocity and acceleration via curve fitting (see Methods, Movies S1, S2, S3, S4, S5, S6, S7, S8, S9 and Supporting Information Note S4). Though both velocity and acceleration changed significantly with time, under the same release rate, they showed a consistent curve across all capillaries (Figure S7). Therefore, we employed the maximum velocity *V*_max_ and maximum acceleration *A*_max_ as representative parameters to characterize the flow motion. As the rates of uptake and release varied from 1 to 10 to 100 mL·min^–1^, the maximum speeds and accelerations of the water fronts increased in the range of 22–360 mm·s^–1^ and 80 to 3000 mm·s^–2^, respectively (Table S2). The measured charge exhibited a quasi-linear dependence with respect to *V*_max_ and *A*_max_ on the semilog scale, suggesting a power-law relationship (Figure 4b,c).

Qualitative descriptions of charge linearly increasing with flow rate are common in the streaming currents literature.^64,65,69^ This observation is well-described by combining the Poiseuille flow with the electrical double layer theory.^64,65,70,71^ In the light of these models, one would expect that in our experiments the total charge carried by the liquid should remain constant regardless the release rate (Supporting Information Note S5). However, our laboratory results diverge dramatically: the dispensed charge (*Q*_4_) through hydrophobic capillaries scale linearly with the logarithm of the maximum flow speed or acceleration. We attribute it to the transient nature of our pipetting experiments, entailing a finite liquid volume and unsteady flow, which prevent the establishment of a steady streaming-current regime; also, the macroscopic fluid motion may be nonlinearly coupled with charge distribution within the electrical double layer. Development of a quantitative model to explain these observations falls beyond the scope of this report. We close the section with the following question: while *Q*_2_ and *Q*_4_ are equal and opposite in magnitude, why are they dependent on the release rate but not on the uptake rate?

### Charge Conservation Across Sequential Pipetting Cycles

To answer the question raised above, we systematically examined charge conservation beyond a single pipetting cycle. Sequential pipetting experiments were performed with varying combinations of liquid uptake and release rates. Figure 5 shows representative results obtained using an ODTS-coated capillary over 15 continuous cycles. For cycles *n* = 1–5, the uptake and release rates were set to 1 mL·min^–1^, yielding values of *Q*_2_ (−0.45 ± 0.03 nC) and *Q*_4_ (0.45 ± 0.02 nC). For cycles numbered *n* = 6–10, the uptake rate was increased to 100 mL·min^–1^, keeping the release rate fixed at 1 mL·min^–1^. *Q*_2_ and *Q*_4_ became −0.47 ± 0.02 nC and 0.44 ± 0.02 nC, largely unchanged and as we demonstrated before. In contrast, when the release rate was increased to 100 mL·min^–1^ (with uptake rate lowered to 1 mL·min^–1^) at cycle *n* = 11, *Q*_4_ responded immediately to 0.67 nC, whereas *Q*_2_ remained to be −0.47 nC. As cycles repeated with the new release rate, at *n* = 12, *Q*_2_ changed to −0.66 nC in a way became the exact opposite of *Q*_4_. This delayed response indicates that the droplet release rate (*Q*_4_^*n*–1^) influences the solid surface charge distribution, which becomes apparent during the subsequent uptake process (*Q*_2_^*n*^). N.B.: the superscript denotes the cycle number.

**Figure 5:**
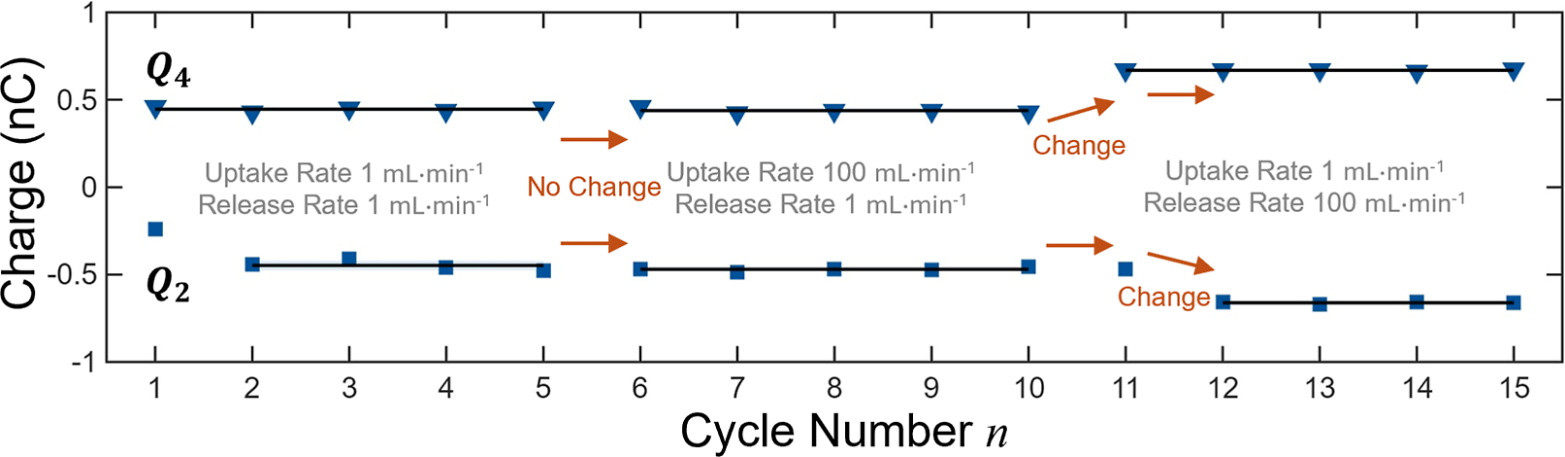
Inter cycle charge conservation in sequential pipetting. Charges *Q*_2_ (uptake) and *Q*_4_ (release) recorded for 15 consecutive cycles for an ODTS-coated capillary. Three combinations of uptake/release rate tested as indicated in the figure. The arrows (→) highlight the charge response to the rate change. Changes in the uptake rate had no effect on *Q*_2_ and *Q*_4_ (*n* = 6). When the release rate is increased from 1 to 100 mL·min^–1^ at cycle *n* = 11, *Q*_4_ rises immediately while *Q*_2_ follows in the next cycle. Mean value (black line) and standard deviation (shaded area, not obvious due to a small value) relating to stable results were presented. Because there is no release history before uptake at *n* = 1, the first *Q*_2_ value is different from the 2nd to 5th and was not included in calculating mean and standard deviation.

Similar trends were also observed for the APTES (2 s)-ODTS and the APTES (5 s)-ODTS surface treatments (see Supporting Information Note S6). These observations suggest that charge conservation in sequential pipetting is more appropriately formulated across cycles, rather than strictly within an individual cycle. We therefore propose the following intercycle charge conservation relation for sequential pipetting

1 $$Q_{4}^{n-1}+Q_{1}^{n}+Q_{2}^{n}+Q_{3}^{n}=0$$

For comparison, the conventional charge conservation relationship for a pipetting experiment is written as

2 $$Q_{1}^{n}+Q_{2}^{n}+Q_{3}^{n}+Q_{4}^{n}=0$$

To evaluate which formulation better describes the mechanism, we define the following charge residuals

3 $$r_{inter}=Q_{4}^{n-1}+Q_{1}^{n}+Q_{2}^{n}+Q_{3}^{n}$$

4 $$r_{intra}=Q_{1}^{n}+Q_{2}^{n}+Q_{3}^{n}+Q_{4}^{n}$$

A simple manipulation establishes the difference between the residuals

5 $$r_{intra}-r_{inter}={\Delta Q_{4}=Q}_{4}^{n}-Q_{4}^{n-1}$$

To establish which of the two charge conservation formulations—intercycle or intracycle—captures sequential pipetting accurately, we designed a new experiment, wherein the release rates were changed continuously from one pipetting cycle to the next. Specifically, the water uptake rate was fixed at 10 mL·min^–1^, while three release rates (1, 10, and 100 mL·min^–1^) were employed, resulting in six types of rate changes between successive tests. Three corresponded to the following serial step-ups (1 → 10, 10 → 100, and 1 → 100) followed by the following step-downs (100 → 10, 10 → 1, and 100 → 1). This pattern was repeated 10-times, leading to 60 consecutive test cycles for the three types of capillaries, affording us with an experimental data set with continuously varying release rates to compare the accuracy of the intercycle formulation with that of the intracycle formulation at describing charge conservation.

Figure 6a–c presents the comparisons of the distributions of the intercycle residual, *r*_inter_, with the intracycle residual, *r*_intra_, for each surface treatment; also, the measured values of Δ*Q*_4_ are plotted as mean ± standard deviation above the histogram. What stands out is that *r*_inter_ follows a bell-curve narrowly centered around zero, whereas *r*_intra_ follows a bimodal distribution that flaunts a minimum around zero (not a maximum). This demonstrates that *r*_inter_ is agnostic to the release rate, whereas *r*_intra_ is extremely sensitive to it. In other words, if sequential release rates are similar, then Δ*Q*_4_ → 0 and the two residuals overlap, i.e., *r*_inter_ = *r*_intra_ = 0. However, when the sequential release rates are dissimilar (the case in this experiment), then Δ*Q*_4_ diverges from zero and so does *r*_intra_ (see eq 5 above).

**Figure 6:**
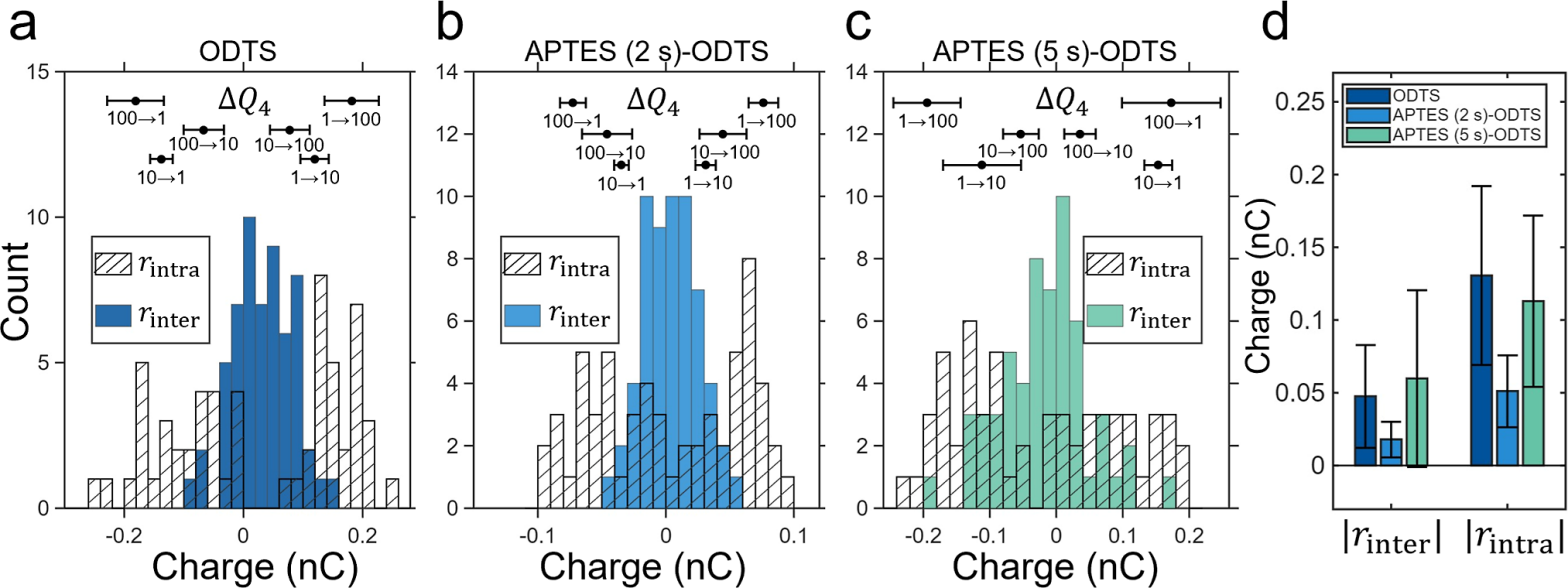
Distributions of the intercycle residual *r*_inter_ (filled bars) and the intracycle residual *r*_intra_ (hatched bars) for (a) ODTS, (b) APTES (2 s)–ODTS, and (c) APTES (5 s)–ODTS capillaries. The uptake rate was fixed at 10 mL·min^–1^, while the release rate was varied in a symmetric cyclic sequence (1 → 10 → 100 → 10 → 1 → 100 → mL·min^–1^), resulting in approximately 60 consecutive cycles for each chemical makeup. Data points indicate the six types of changes in the release charge (Δ*Q*_4_). Across all surfaces, *r*_inter_ remains narrowly distributed around zero, whereas *r*_intra_ exhibits a much broader spread and frequently aligns with the Δ*Q*_4_. Note: Refer to Supporting Information Table S3 for the absolute values of Δ*Q*_4_ for the various scenarios. (d) Absolute mean values (±1 standard deviation) of the intercycle and intracycle charge residuals for the three surface treatments. The consistently smaller |*r*_inter_| across all capillaries indicates that charge conservation is more accurately captured when formulated across successive wetting–dewetting cycles, especially under varying release rates.

It is important to note that *r*_intra_ distributes approximately symmetrically following Δ*Q*_4_ in a bimodal distribution around zero because the release-rate changes symmetrically in our experiment (including both increases and decreases). By calculating the mean values and standard deviations with the absolute values of *r*_intra_ and *r*_inter_, it is evident that the intercycle formulation captures charge conservation more accurately than the intracycle formulation (Figure 6d). The broader spread of *r*_intra_, together with its alignment with Δ*Q*_4_, indicates that this residual mainly arises from how the measurement cycle is defined, rather than from a failure of charge conservation.

The intercycle conservation further explains two experimental observations, where the effects of uptake and release flow rates were recorded on electrification (Figures 2 and 3). The release rate directly modifies *Q*_4_^*n*^, while *Q*_2_^*n*^ remains unchanged because the uptake step has already occurred; charge conservation is subsequently recovered through the adjustment of *Q*_2_^*n*+1^ in the following cycle. In addition, a change in the charge distribution at the solid surface after liquid release not only changes *Q*_2_ at the next cycle, but may also influence the *Q*_1_as the tip contacts liquid surface, resulting in a greater scatter in *Q*_1_ compared to *Q*_3_ as the capillary detaches from the water.

We close this section by noting that charge conservation during wetting–dewetting cycles reconciled via intercycle formulation implies that during the dewetting step, the surface gets altered—either via charge accumulation/shortage and/or molecular rearrangement—which controls the amount of charge acquired during wetting in the subsequent cycle. Quantitative insights into the surface modification as well as the nature of the surface charge of common hydrophobes^21^ warrants concerted experimental and theoretical investigation.

## Conclusion

We unveil an intercycle coupling in water–hydrophobe electrification that emerges under repeated wetting–dewetting conditions. Using controlled pipetting experiments, we demonstrate interfacial charging is governed by liquid–solid separation kinetics during the release step, whereas the kinetics of liquid–solid contact formation during uptake play no measurable role. As a result, electrification during the release step of a given cycle directly influences charge acquisition during the uptake step of the subsequent cycle. To account for this behavior, we introduce an intercycle formulation of charge conservation that provides a physically consistent description of charge balance during wetting–dewetting under varying flow conditions. This framework robustly describes laboratory observations across flow rates spanning 3 orders of magnitude and across water–hydrophobe interfaces exhibiting negative, near-neutral, and positive surface charge densities. Together, these findings establish liquid–solid separation as the dominant control parameter for electrification in wetting–dewetting cycles and highlight the inherently history-dependent nature of interfacial charging. These insights are relevant to a wide range of natural and technological processes, including droplet motion on smooth/rough hydrophobic surfaces, liquid-handling operations, self-assembly at aqueous interfaces, and micro- and nanofluidic systems.

## Materials and Methods

### Sample Preparation

Cylindrical capillaries (borosilicate glass, 1.5 to 1.8 × 100 mm, Kimble Chase, Cat. No. 34500-99) were rinsed with acetone and Milli-Q water, followed by hydroxylation (surface activation) with a fresh-made piranha solution (H2SO4: H2O2 = 4:1, v/v) at about 70 °C for 30 min. After activation, the samples were rerinsed with Milli-Q water and dried in a vacuum oven at room temperature for about 1 h. Upon cooling, samples were subjected to one of three silanization treatments.

- Treatment 1 (ODTS only): samples were immersed in trichloro­(octadecyl)­silane (ODTS) for 5 min.
- Treatment 2 (APTES (2 s)-ODTS): samples were briefly exposed to (3-aminopropy)-triethoxysilane (APTES) for 2 s, rinsed with toluene, and immersed in ODTS solution for 5 min.
- Treatment 3 (APTES (5 s)-ODTS): this treatment was the same as Treatment 2, but with an APTES exposure time of 5 s.

All silanization treatments were performed in 20 mL of freshly prepared solution (toluene to silane = 400:1, v/v) at about 20 °C. Then, they were rinsed using toluene and ethanol, dried in the vacuum oven, and stored in sealed glass test tubes overnight before testing. Capillaries were glued to 20G*1″ Monoject needles (Kendall) to allow connection to tubing or pipettes.

### Charge Measurement

A brass Faraday cup was employed for the measurement, comprising an outer cylinder (8 cm in diameter and 12 cm in height) and an inner cylinder (6 cm in diameter and 10 cm in height), separated by dielectric foam. The cup was connected to an ultrasensitive electrometer (Keithley 6517B) with a detection limit of 10 fC (Figure 1). The capillaries were mounted on a holder that allowed manual up-and-down movement and were connected to a PHD ULTRA syringe pump (Harvard Apparatus) via a polytetrafluoroethylene (PTFE) capillary tube, enabling precise liquid uptake and release.

Before measurements, the Faraday cup was connected to the electrometer and left overnight to stabilize the system. For each trial, 50 μL of Milli-Q water (pH ≈5.6) was introduced into the capillary (total volume about 90 μL). The water was contained within the glass capillaries and never came into contact with the tubing or syringe, avoiding unwanted contamination. When the water was dispensed from the capillary into the Faraday cup, the transferred charge was captured and recorded using LabVIEW software.

### Velocity Measurement

A high-speed camera (Phantom v1212, with a Nikon 55 mm f/2.8 Micro Nikkor Lens) was employed to film the dispensing process at a resolution of 384 × 288 pixels. A backlit LED source enhanced the contrast. The frame rate was set between 100 and 3000 fps, depending on the dispensing rate. A ruler was employed for calibration, and the spatial resolution was determined to be 3.69 μm/pixel. Image sequences were analyzed using MATLAB (Supporting Information Note S2).

## Supplementary Material





































## Data Availability

All data for evaluating the conclusions in this paper are present in the main text or Supporting Information.

## Notes

It describes the surface characterization, experimental details for the uptake/release rates, high-speed imaging, postprocessing, and the limitations of classical streaming current models in finite-volume capillary flows; high-speed imaging movies showing representative liquid release dynamics on three surface chemistries (ODTS, APTES (2 s)–ODTS, and APTES (5 s)–ODTS) at three dispensing rates (1, 10, and 100 mL·min^–1^) are provided (Movies S1–S9, one representative measurement for each condition); also, excel files containing the numerical data used to generate Figures 1–6, Figures S3, and S8 are included (PDF)
ODTS_rate1_100fps_Sample.mp4 (MP4)
ODTS_rate10_1000fps_Sample.mp4 (MP4)
ODTS_rate100_3000fps_Sample.mp4 (MP4)
APTES2s_rate1_100fps_Sample.mp4 (MP4)
APTES2s_rate10_1000fps_Sample.mp4 (MP4)
APTES2s_rate100_3000fps_Sample.mp4 (MP4)
APTES5s_rate1_100fps_Sample.mp4 (MP4)
APTES5s_rate100_3000fps_Sample.mp4 (MP4)
APTES5s_rate10_1000fps_Sample.mp4 (MP4)
Fig 1.xlsx (XLSX)
Fig 2.xlsx (XLSX)
Fig 3.xlsx (XLSX)
Fig 4.xlsx (XLSX)
Fig 5.xlsx (XLSX)
Fig 6.xlsx (XLSX)
Fig S3.xlsx (XLSX)
Fig S8.xlsx (XLSX)
