# Supplementary material for: It is Separation, Not Contact: Electrification at Water–Hydrophobe Interfaces during Wetting–Dewetting Cycles
Source: Langmuir. 2026 Jan 30;42(5):4010–9. doi: 10.1021/acs.langmuir.5c05487 (PMC12895528; doi:10.1021/acs.langmuir.5c05487)
Supplement: Supplementary file 1 [file la5c05487_si_001.pdf]

Supporting Information

It is Separation, Not Contact: Electrification at Water–Hydrophobe Interfaces during Wetting-Dewetting Cycles

Yinfeng Xu*,†,‡,§ & Himanshu Mishra*,†,‡,§

†Environmental Science and Engineering (EnSE) Program, Biological and Environmental Science and Engineering (BESE) Division, King Abdullah University of Science and Technology (KAUST), Thuwal, 23955-6900, Saudi Arabia

‡Sustainable Food Security Center of Excellence, King Abdullah University of Science and Technology (KAUST), Thuwal 23955-6900, Saudi Arabia

§Interfacial Lab (iLab), King Abdullah University of Science and Technology (KAUST), Thuwal 23955-6900, Saudi Arabia

*Email: yinfeng.xu@kaust.edu.sa
*Email: himanshu.mishra@kaust.edu.sa

This PDF file includes the following:

Supporting Information:

SI Note S1: Surface Characterization and an Explanation for Interfacial Charge Reversal
SI Note S2: Experiment Details for Uptake/Release Rate
SI Note S3: Uptake/Release Rate on Uptake Charge
SI Note S4: High-Speed Imaging and Postprocessing
SI Note S5: Limitations of Classical Streaming Current Models in Finite-Volume Capillary Flows
SI Note S6: Charge Response in Sequential Pipetting for APTES-Modified Capillaries.

SI Note S1: Surface Characterization and an Explanation for Interfacial Charge Reversal

To characterize the surface quality, silicon wafers (Silicon Valley Microelectronics, p-type <100>) were cut into pieces (1×6 cm^2) and underwent the same treatment as glass capillaries. On the wafer samples, the advancing and receding contact angles were measured (details in Table S1 below). Surface topography was characterized using atomic force microscopy (AFM), and root mean square (RMS) roughness was calculated (Table S1).

Contact Angle Measurement. The advancing and receding angles were determined using the Krüss drop shape analyzer 100. The angles were measured by adding 10 μL to a 2 μL droplet and then removing it at a rate of 0.2 μL s^{-1}. For each wafer sample, at least three points were tested, and four measurements for advancing and receding angles were made during each advancing-receding cycle (Table S1). The recorded images were analyzed using *Advance* software (Krüss GmbH), with a manual baseline and the tangent fitting method for the droplet shape. The treated surfaces were hydrophobic (Table S1).

Surface Topography. In addition, AFM imaging was performed using a JPK Nanowizard Ultraspeed II in tapping mode with silicon probes (tip radius 8 nm, spring constant 2.8 N/m, and frequency 75 kHz). The topography data were processed using Gwyddion software, including first-order flattening and RMS roughness calculation (Figure S1).

Table S1. Contact angles and surface roughness for the samples

Coatings	Advancing angle (°)	Receding angle (°)	RMS roughness (nm)
ODTS	121 ± 1	92 ± 3	1.2
APTES (2 s)-ODTS	117 ± 2	97 ± 2	1.7
APTES (5 s)-ODTS	117 ± 2	96 ± 2	0.9

Abbreviations: ODTS, octadecyltrichlorosilane; APTES, (3-aminopropyl)triethoxysilane.

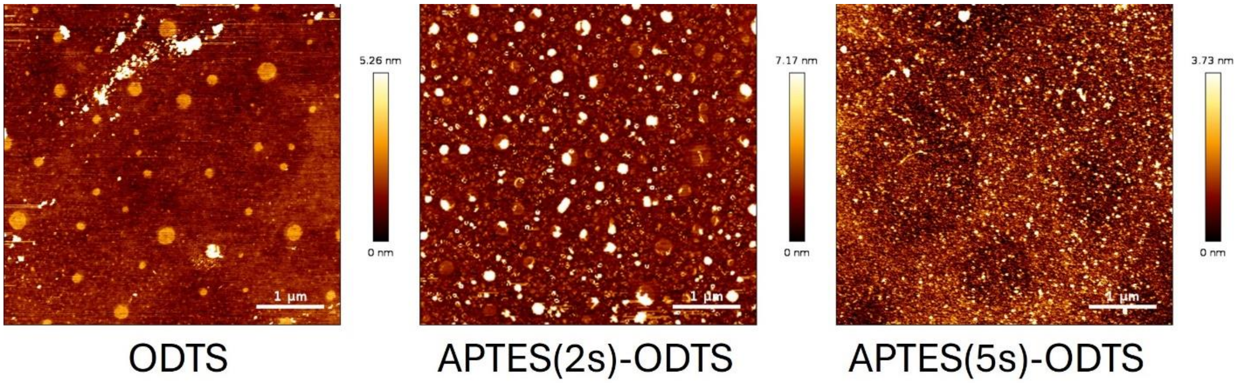

Figure S1. Atomic force microscopy imaging for a wafer with three treatments.

An explanation for Surface Charging of ODTS and ODTS/APTES in Water

For ODTS-coated capillaries, the observation of positively charged droplets is consistent with the well-known behavior of hydrophobic surfaces such as alkylsilane, PDMS, and fluoropolymers. Although this charging phenomenon is robust and widely reported, its microscopic origin—whether arising from surface-bound electrons, specific ion adsorption, or other mechanisms—remains unresolved. Our previous work (Nauruzbayeva et al.[1]) showed that common hydrophobic interfaces carry an intrinsic negative charge in air. This charge is presumably due to surface-bound electrons, present in "defect sites" (see Lowell et al.[2-4] and Bard et al.[5, 6]). This interpretation explains the electrification at the ODTS–water interface. That is, as water is drawn into the negatively charged ODTS capillary, it draws excess cations (predominantly H_3O^+ in the case of pH-neutral water) from the reservoir, stabilized/residing within the electric double layer at the capillary–water interface. As the water is dispensed, these excess cations are responsible for the positive charge carried by the pendant drop into the electrometer (Fig. 1b-c).

In contrast, the introduction of APTES neutralizes or even reverses the nature of the electrification at the water–hydrophobe interface. We attribute it to the primary amines that get protonated to –NH_3^+ as they contact water (pH = 5.6). In this scenario, the capillary–water interface acquires a net positive charge, which can be experienced at microscopic distances (Debye length [illegible]). This is responsible for drawing excess OH^- ions from the reservoir, as explained above, and causing the dispensed droplets to carry a net negative charge (i.e., charge reversal). Here, we also want to comment on the extreme sensitivity of the electrification of an ODTS surface to the APTES content. Presumably, it's because the background (negative) charge presented by ODTS is fairly low (–3.1×10^{-6} $C\cdot m^{-2}$ = [illegible] electronic charge every square nanometer), while each APTES molecule presents a unit positive charge. Therefore, tiny enhancements in the APTES content have dramatic effect on the surface charge density. To account for this during our sample preparation, the surface concentration of APTES was controlled tightly by quenching the silanization reaction (see Methods Section for details).

SI Note S2: Experimental Details for Uptake/Release Rate

To better understand the detailed charge transfer during the complete liquid handling cycle, the charge was monitored and analyzed across four stages of the process (Figure S2a). In each experiment, water was initially placed in the Faraday cup. Figure S2b presents a representative charge transfer profile for an ODTS-coated capillary operated at a low flow rate of 1 mL·min^{-1} for the liquid uptake and release. The light blue curve represents the raw data, whereas the dark blue curve presents the data after the drift correction.

Stage 1: Contact (t_1–t_2). The capillary was empty and brought into contact with the water surface in the Faraday cup. A small negative charge was detected, which is attributed to the naturally negative surface potential of the ODTS-treated capillary.

Stage 2: Liquid uptake (t_2–t_4). At this stage, 50 µL of water was drawn into the capillary using the syringe pump. During this period, the measured charge gradually became more negative, reflecting the accumulation of a charge during capillary filling.

Stage 3: Capillary lift (t_4–t_5). The capillary was manually lifted and suspended above the Faraday cup. A small positive charge was observed during this stage.

Stage 4: Liquid release (t_5–t_7). Water was dispensed back into the Faraday cup at the same low rate of 1 mL·min^{-1}. It took approximately 25 s to empty the capillary completely. Between t_5 and t_6, the charge signal remained stable with no significant increase or decrease. This plateau is likely due to two factors: mechanical backlash in the syringe pump and the slow-release rate. The water formed pendant droplets at the capillary tip, detaching once the weight exceeded the adhesion force. At the moment of detachment (around t_6), a clear positive charge step was observed.

At this low release rate, the 50 µL volume typically formed two distinct droplets before the capillary was emptied, resulting in two charge steps visible between t_6 and t_7. At higher release rates, the entire volume was released at once, producing only one charge step during the release stage. The total duration of the liquid release stage varied significantly with the release rate: approximately 40 s at 1 mL·min^{-1}, compared to about 20 s at 100 mL·min^{-1} (including the pump backlash delay).

Drift and Correction. A slow, consistent positive drift was observed in all raw charge data over the measurements. This drift is likely due to electrochemical interactions between water and the brass walls of the Faraday cup, as well as the presence of dissolved oxygen and carbon dioxide under ambient conditions.

To correct for this drift, the period (t_5–t_6), during which the capillary was held above the cup and no liquid transfer occurred, was employed as the baseline reference. The linear fit of the charge signal over this interval was extrapolated across the entire dataset and was subtracted to yield the corrected charge curve (dark blue in Figure S2b). This approach reliably removed background drift and was consistently applied across all measurements.

We analyzed the baseline drift across 149 experimental runs, including various capillary types, uptake rates, and release rates. Figure S2c reveals that the measured drift remained minimal throughout the tests, with a mean value of 2.3 pC·s^{-1} and a standard deviation of 0.3 pC·s^{-1}. This narrow spread indicates excellent consistency, suggesting that the drift is small in magnitude and robust across the experimental variations. These results validate the reliability of the subsequent

charge measurements and confirm that baseline drifts have a negligible influence on the data interpretation.

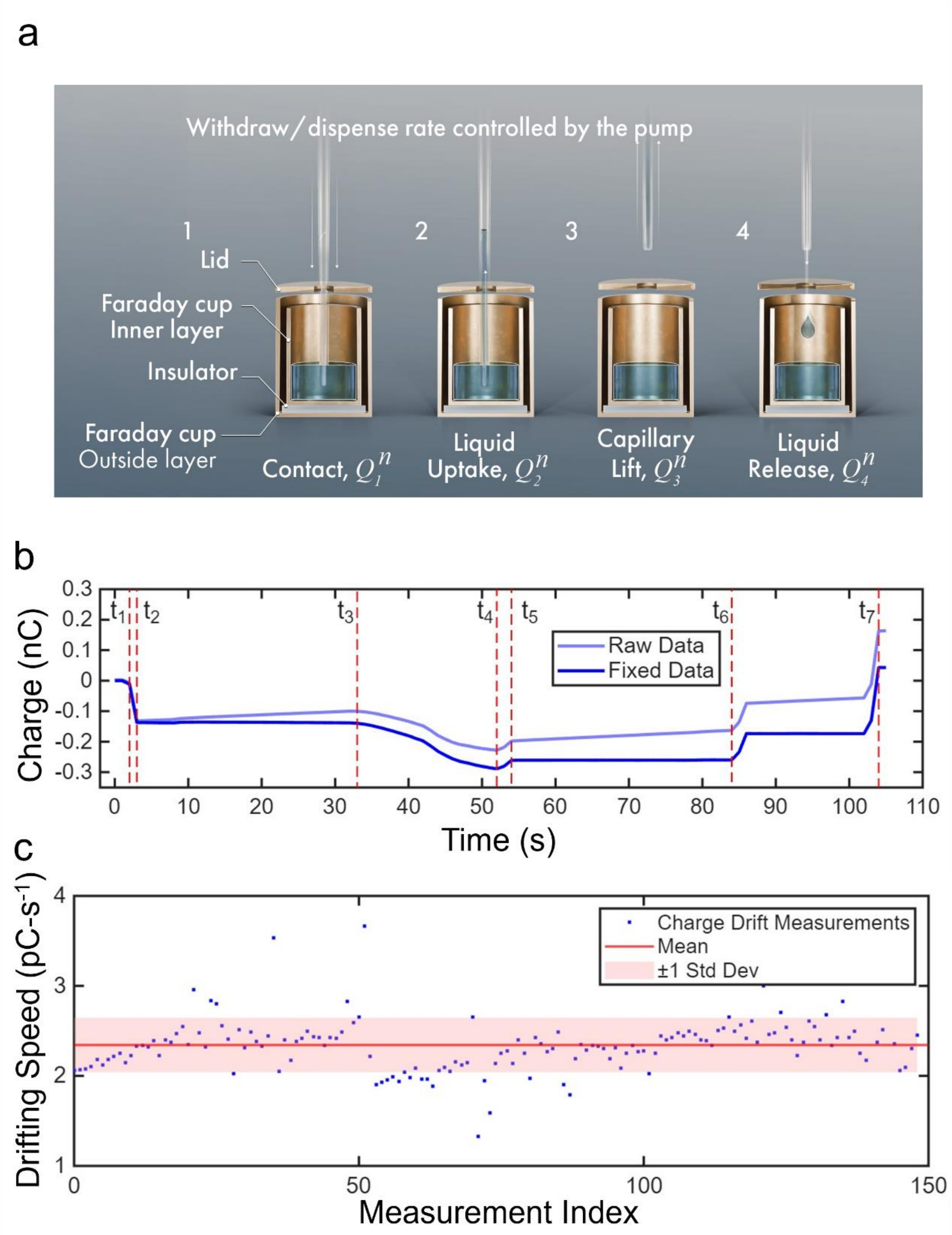

Figure S2. Charge measurement and drift correction. **(a)** Schematic of the four-stage liquid handling cycle (reproduced from Figure 2a in the main text). Presented to facilitate the stage-specific charge signal analysis presented in Figure S2b. **(b)** A typical result during the four steps:

t_1–t_2: contact, t_2–t_4: liquid uptake, t_4–t_5: capillary lift, and t_5–t_7: liquid release. The light blue curve marks the raw data, which were corrected for drift to produce the dark blue fixed data curve with accurate values for each stage. The sample in (b) is dispensed using an ODTS capillary at a low rate of 1 mL·min^{-1} for withdrawal and release. **(c)** Drift measured from 149 tests by capillary and flow rate, revealing a small and consistent value of 2.3 ± 0.3 pC·s^{-1}.

SI Note S3: Uptake/Release Rate on Uptake Charge

Result for the uptak/release rate effect on uptake charge Q_2 (Figure S3). Like Q_1 Q_2 increases as release rate increases, while the uptake rates have no significant influence. In summary, only the release rate influences Q_2 and Q_1. Note, these results are based on averaged value of multiple measurements.

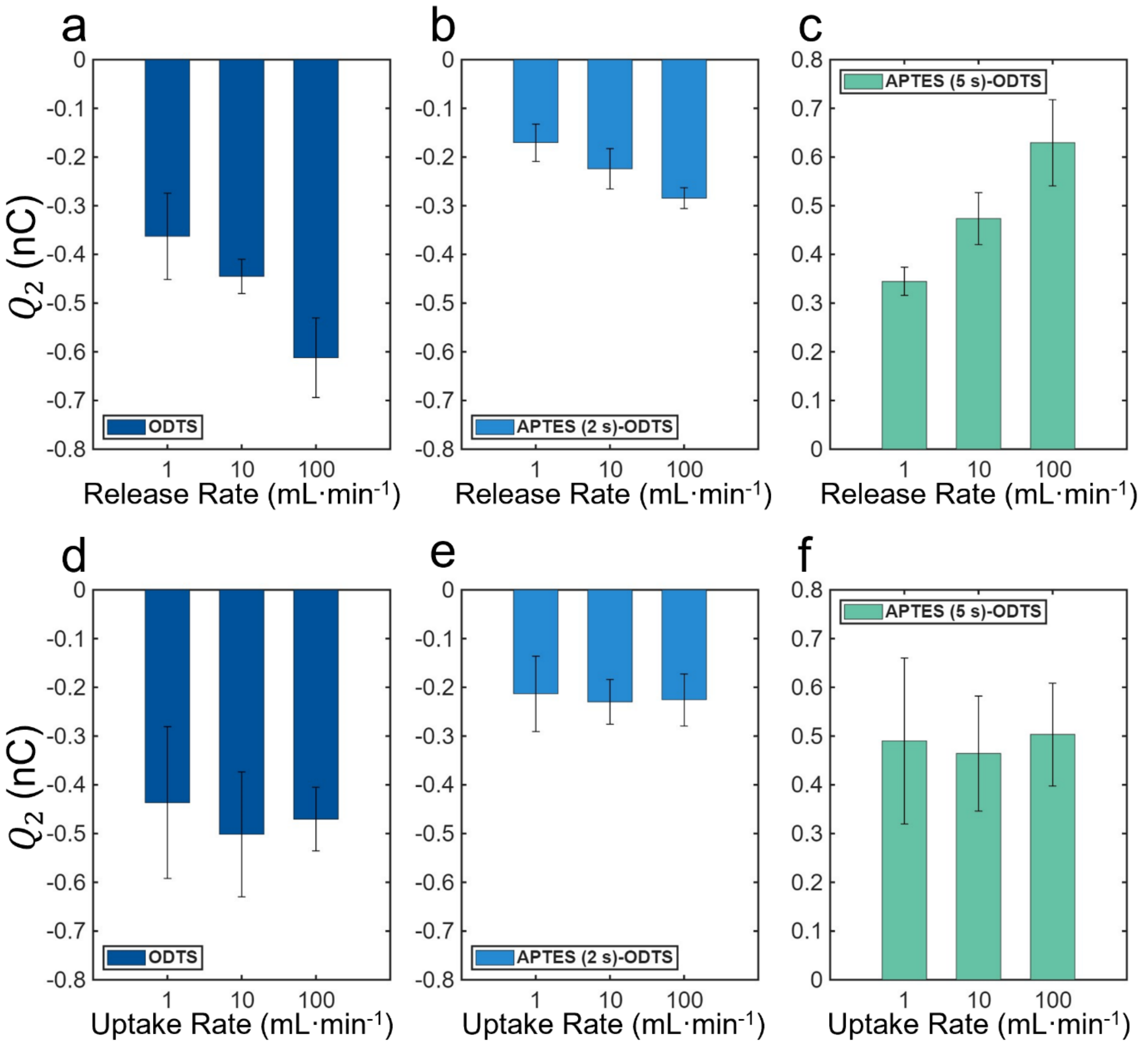

Figure S3. Effect of uptake/release rate to the uptake charge Q_2 carried by 50 µL water. **(a-c)** Charge Q_2 measured during liquid release as a function of the release rate for capillaries coated with **(a)** ODTS, **(b)** APTES (2 s)–ODTS, and **(c)** APTES (5 s)–ODTS, respectively, demonstrating an irrefutable effect. **(d-f)** As the liquid uptake rate increases, the measured Q_2 reveals no significant difference for the capillaries. Note: ODTS, octadecyltrichlorosilane; APTES, (3-aminopropyl)triethoxysilane.

SI Note S4: High Speed Imaging and Postprocessing

To extract quantitative information on water interface displacement from high-speed imaging data, we developed a custom MATLAB script that processes 8-bit grayscale TIFF images recorded at known frame rates. The image processing algorithm was based on background subtraction and binary segmentation to extract the vertical motion of the water interface. A static background was generated by averaging the first five frames, assuming negligible initial movement. Each following frame was subtracted from this background to isolate the motion-induced changes. The pixel contrast was enhanced, and a fixed threshold was applied to produce a binary mask of moving regions. Morphological operations (closing and hole filling) and area-based filtering were employed to suppress noise and retain physically meaningful features. The vertical displacement of the interface in each frame was quantified as the maximum vertical extent of the foreground region. This simple yet robust method suppresses noise and captures the spatiotemporal dynamics of the interface (see Figure S4).

Each displacement curve was smoothed using a median filter to suppress local spikes while preserving the overall trends. To improve the physical interpretability, nonphysical fluctuations prior to motion onset and after peak displacement were suppressed by isolating the active phase and enforcing a monotonic (nondecreasing) constraint. All traces were temporally aligned using the threshold-crossing point marking the onset of motion and were interpolated onto a common time grid for ensemble averaging (Figure S5).

High-speed imaging revealed that, at release rates of 1 and 10 $mL \cdot min^{-1}$, the 50 µL liquid volume was discharged from the capillary in two distinct droplets (SI Movies 1 and 2). After detachment of the first droplet, a noticeable delay preceded the formation of the second droplet. This delay is likely attributable to the time required to re-establish sufficient pressure to overcome the Laplace pressure associated with a high interfacial curvature. In the displacement-time curves, this delay manifests as a plateau during which the liquid interface remains stationary. Notably, the standard deviation (shaded region in Figures S5 and S6a) increases markedly at the beginning of the movement of the second droplet due to variations in the onset timing across trials, rather than sudden displacement events.

Although two droplets are still formed at 10 $mL \cdot min^{-1}$, the interval between them is less than 1 s. Thus, with the charge measured at 1 Hz, the two-step change in the charge signal cannot be resolved, resulting in a single observed step. In contrast, at 1 $mL \cdot min^{-1}$, the two-step charge response is clearly detected (SI Note 2 and Figure S2b).

The resulting displacement-time signals were processed to derive the velocity and acceleration. To extract smooth and physically meaningful motion profiles, a cubic smoothing spline was applied to the ensemble-averaged displacement data using a high smoothing parameter ($p = 0.999$). This approach produced a continuous trajectory that was differentiated numerically to obtain velocity and acceleration. This work evaluates various smoothing levels (p = 0.90, 0.95, and 0.999) and compares them to the Savitzky–Golay filters (fifth-order polynomials with window sizes of 7, 15, and 37). Although the Savitzky–Golay filtering preserved the displacement signal (Figure S6a), its derivatives were noisy and oscillatory due to poor high-frequency noise suppression (Figure S6b-c).[7]

In contrast, the cubic spline displayed smooth, C^2-continuous curves that yielded cleaner derivative profiles.[8] However, excessive smoothing ($p \leq 0.95$) overly dampened sharp features, reducing the

peak magnitudes and distorting the motion onset. A setting of $p = 0.999$ resulted in an optimal balance, preserving crucial kinematic features while reducing noise. Although this may slightly underestimate the peak velocity and acceleration, uniform application across all trials ensures valid relative comparisons.

All experiments were conducted using a fixed liquid volume of 50 µL and capillaries with identical diameters, resulting in a consistent travel distance of about 40 mm. Under these controlled conditions, the maximum velocity and maximum acceleration were employed as critical metrics to compare the dynamics of liquid motion. Figure S7 presents the corresponding velocity and acceleration profiles, derived from the smoothed displacement data. Table S2 summarizes the extracted peak values.

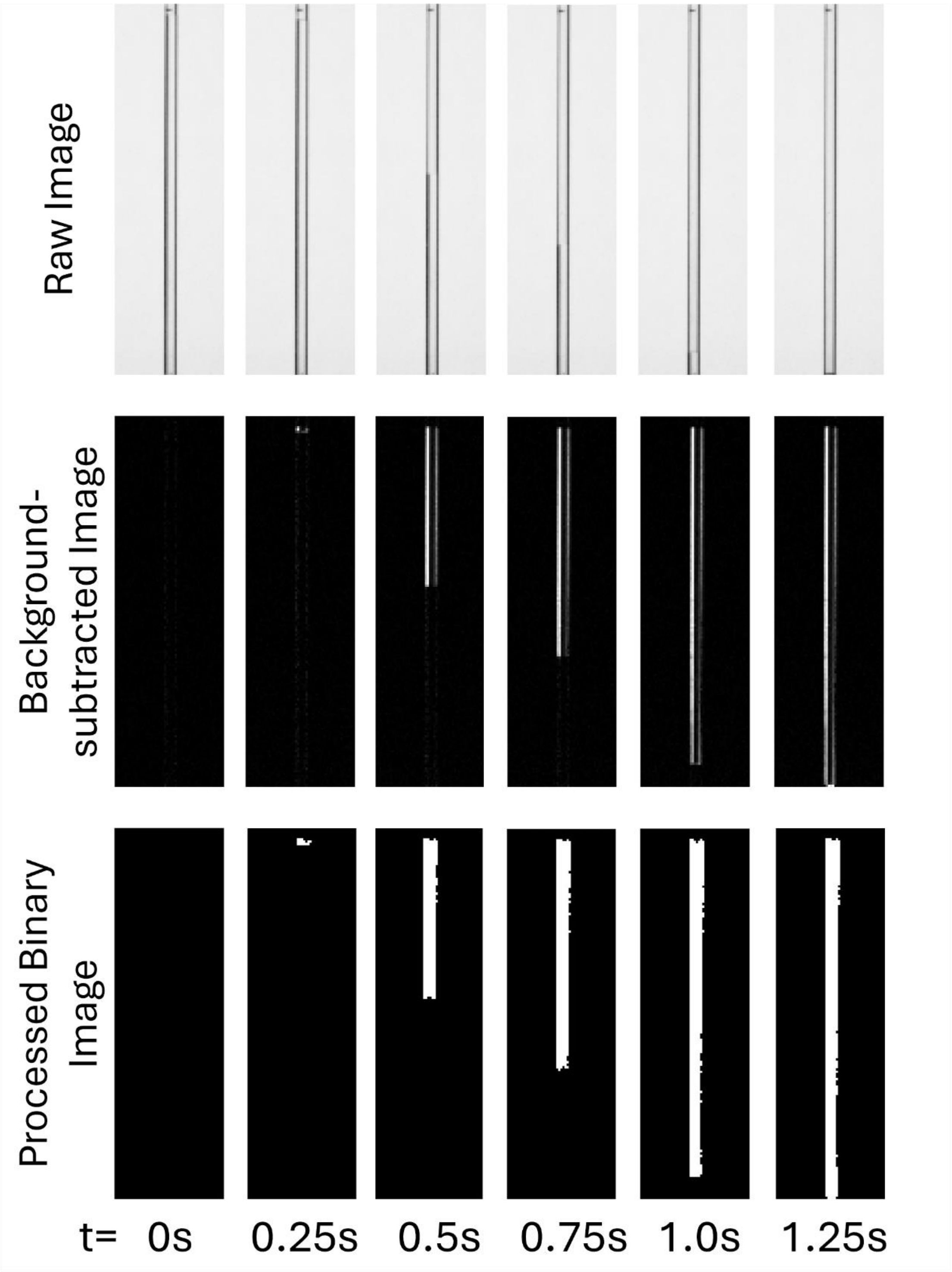

Figure S4. Image processing steps to track vertical water interface motion over time (t = 0–1.25 s). Top row: raw high-speed images. Middle row: background-subtracted images using an average of the initial frames. Bottom row: processed binary images after contrast enhancement, thresholding, morphological filtering, and noise removal. The interface displacement is quantified using the

maximum vertical extent of the detected regions. Example: ODTS-treated capillary, release rate = 10 mL·min^{-1}. Note: ODTS, octadecyltrichlorosilane.

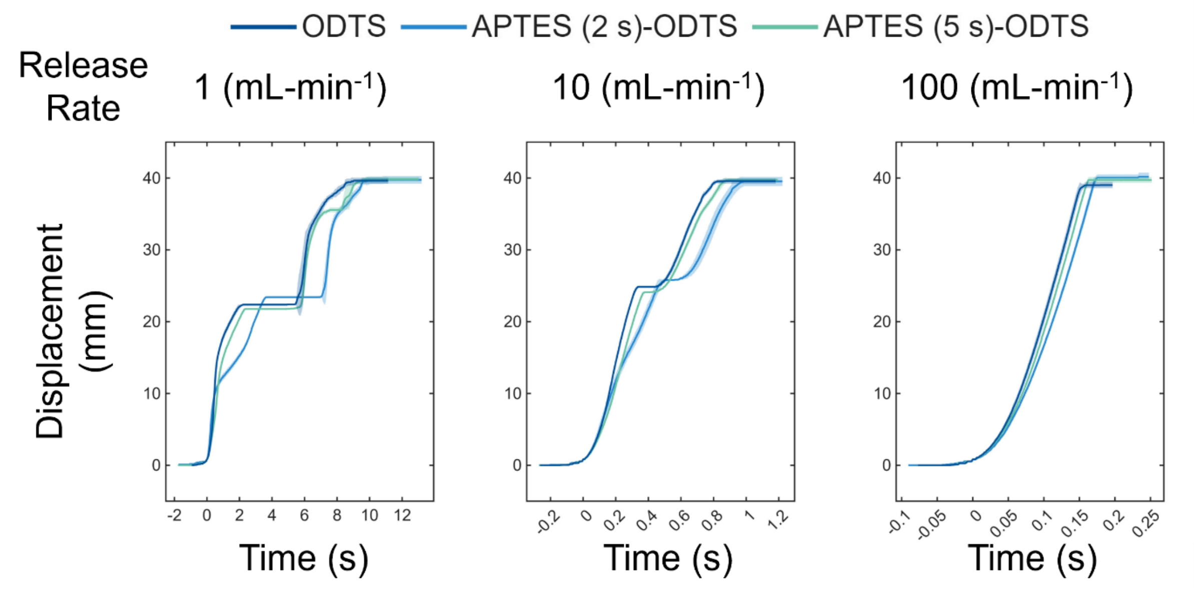

Figure S5. Displacement profiles for three surface treatments (ODTS, APTES (2 s)-ODTS, and APTES (5 s)-ODTS) by release rate (1, 10, and 100). Shaded regions represent the standard deviation across repeated trials for displacement. The displacement curves exhibit minimal variability, highlighting the measurement consistency and robustness of the postprocessing method. Note: ODTS, octadecyltrichlorosilane; APTES, (3-aminopropyl)triethoxysilane.

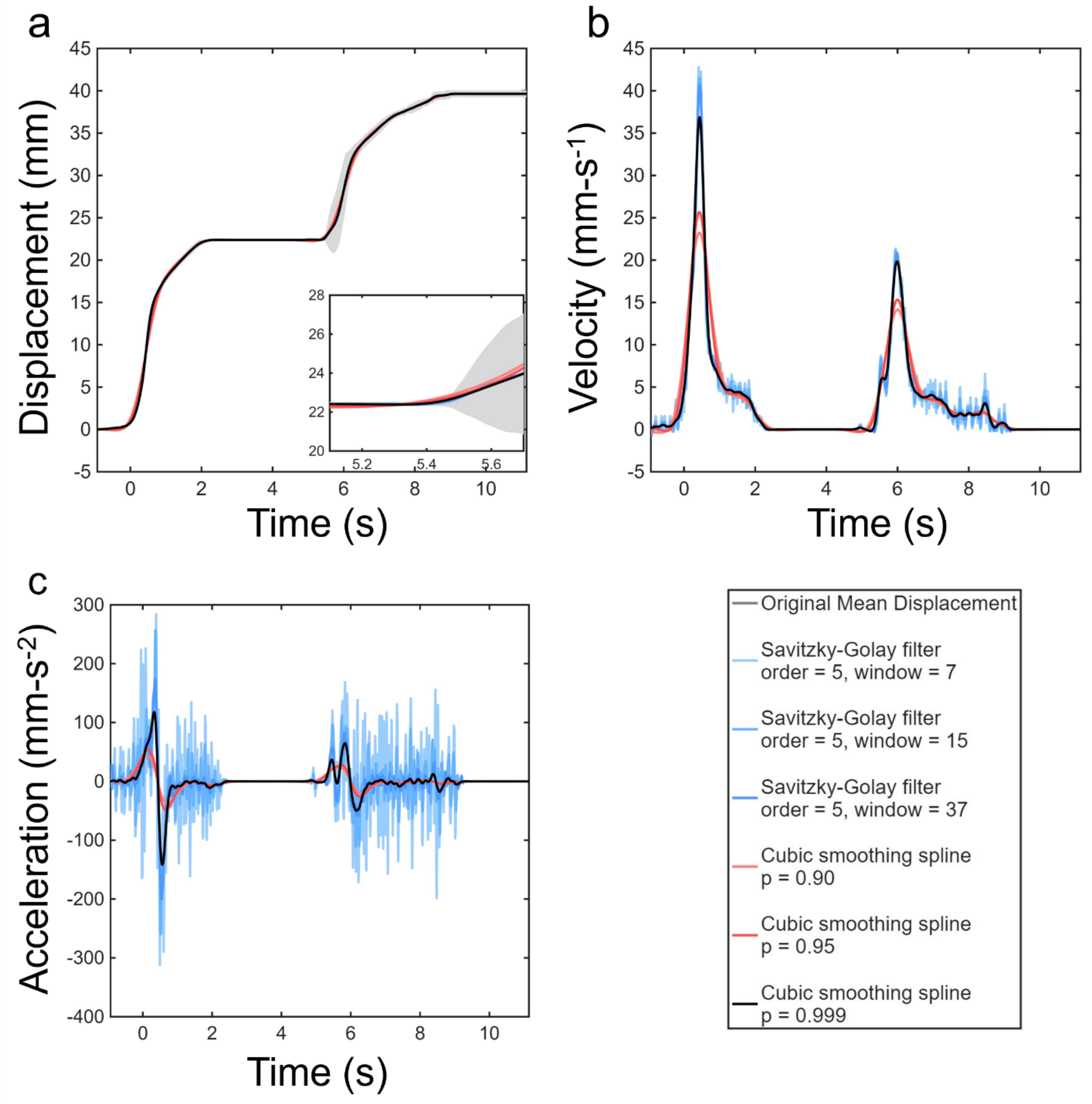

Figure S6. Comparison of smoothing methods applied to **(a)** displacement, **(b)** velocity, and **(c)** acceleration data. Displacement curves were smoothed using cubic splines with smoothing parameters $p = 0.90$, 0.95, and 0.999 (red and black lines), and Savitzky–Golay filters (fifth-order polynomial, window sizes of 7, 15, and 37; blue lines). The original unsmoothed data are in gray. In **(a)**, Savitzky–Golay filters closely match the original displacement data, whereas the lower spline parameters (e.g., $p = 0.90$) introduce artifacts, such as unrealistic rollback near the onset of motion (see inset). In **(b)** and **(c)**, Savitzky–Golay filters fail to suppress noise in velocity and acceleration, whereas cubic spline smoothing (especially at $p = 0.999$) provides cleaner, more physically plausible profiles. Example: ODTS-treated capillary, release rate = 1 mL·min^{-1}.

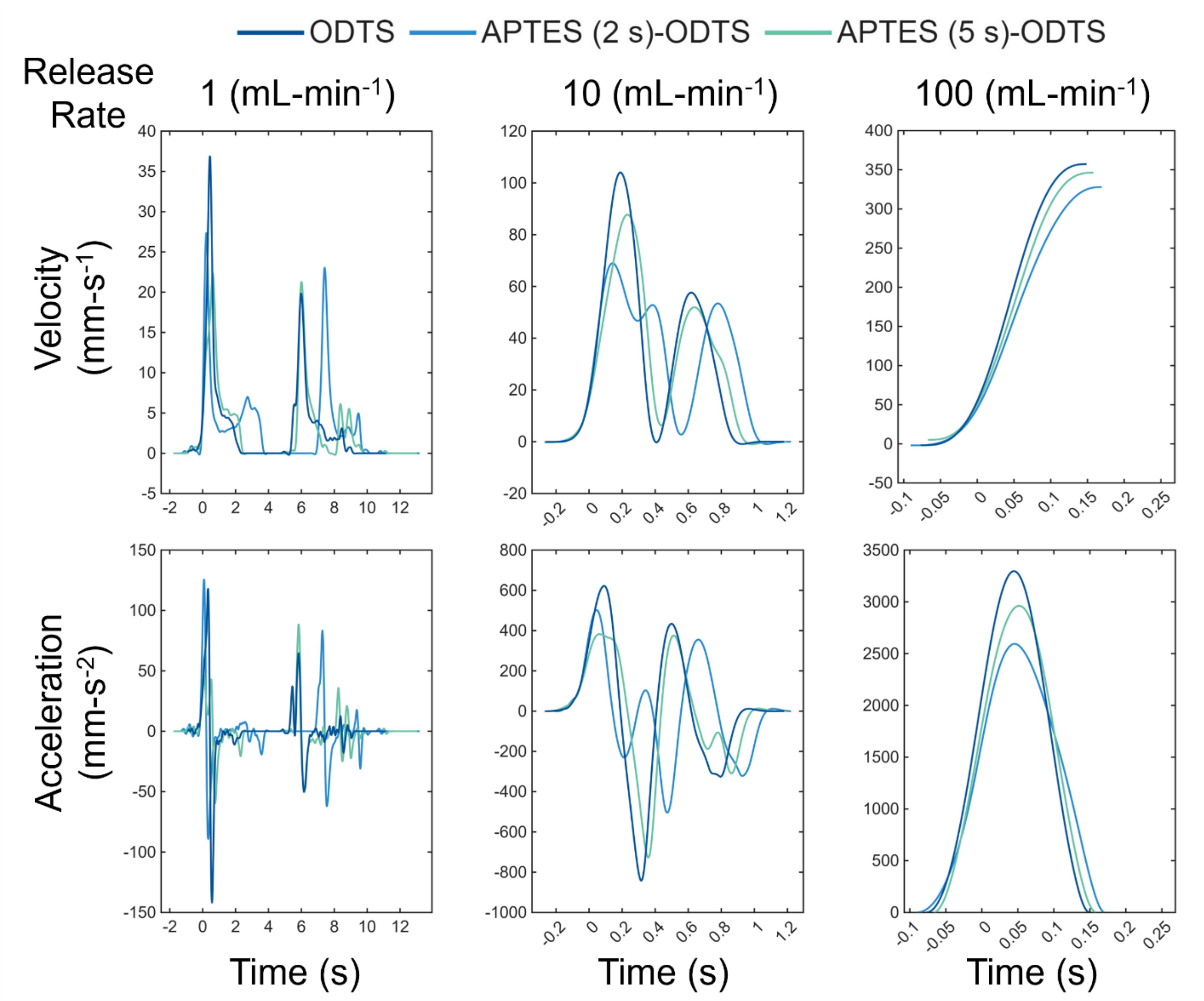

Figure S7. Velocity and acceleration profiles of liquid motion by release rate (1, 10, and 100 mL·min⁻¹) for surfaces treated with ODTS, APTES (2 s)-ODTS, and APTES (5 s)-ODTS. The data were smoothed and differentiated to extract the maximum values of velocity and acceleration, as summarized in Table S2.

Table S2. Maximum Velocity and Acceleration by Release Rate for Treatments

Release Rate ($mL{\cdot}min^{-1}$)		ODTS	APTES (2 s)-ODTS	APTES (5 s)-ODTS
1	V_{max} ($mm{\cdot}s^{-1}$)	36.9	27.3	22.4
	A_{max} ($mm{\cdot}s^{-2}$)	118	126	88
10	V_{max} ($mm{\cdot}s^{-1}$)	104.0	69.0	87.7
	A_{max} ($mm{\cdot}s^{-2}$)	622	502	383
100	V_{max} ($mm{\cdot}s^{-1}$)	357.3	328.0	346.3
	A_{max} ($mm{\cdot}s^{-2}$)	3296	2594	2962

Abbreviations: ODTS, octadecyltrichlorosilane; APTES, (3-aminopropyl)triethoxysilane.

SI Note S5: Limitations of Classical Streaming Current Models in Finite-Volume Capillary Flows

A classical model for streaming current usually combines charge density ρ_e and flow velocity v in a straight cylindrical pipe of radius R and includes the following assumptions:

- Charge density ρ_e, usually described by the Poisson–Boltzmann Equation, depends only on the radial coordinate r (i.e., $\rho_e = \rho_e(r)$) and is time-invariant.
- Flow velocity v may vary with the radial coordinate r and the time t (i.e., $v = v(r,t)$).
- $\rho_e(r)$ and $v(r,t)$ are decoupled.

The current $I(t)$ through the pipe cross section at time t is given by the following:

$$I(t) = \int_0^R \rho_e(r)\, v(r,t) \cdot 2\pi r\, dr$$

Therefore, the total charge Q transported over the time interval $[0,T]$ is

$$Q = \int_0^T I(t)\, dt = \int_0^T \left[\int_0^R \rho_e(r)\, v(r,t) \cdot 2\pi r\, dr\right] dt$$

Interchanging the order of integration yields

$$Q = \int_0^R \rho_e(r) \left[\int_0^T v(r,t)\, dt\right] 2\pi r\, dr$$

The cumulative displacement length of fluid at radial position r is defined as follows:

$$L(r) := \int_0^T v(r,t)\, dt$$

The total charge can be rewritten as follows:

$$Q = \int_0^R \rho_e(r)\, L(r) \cdot 2\pi r\, dr \tag{S1}$$

In this case, the total volume V of liquid dispensed is fixed; thus,

$$V = \int_0^T \left[\int_0^R v(r,t) \cdot 2\pi r\, dr\right] dt = \int_0^R L(r) \cdot 2\pi r\, dr = \text{const} \tag{S2}$$

Combining equations (S8a) and (S8b) yields

$$\frac{Q}{V} = \frac{\int_0^R \rho_e(r)\, L(r) \cdot 2\pi r\, dr}{\int_0^R L(r) \cdot 2\pi r\, dr} \tag{S3}$$

As presented in Equation S3, the ratio of the total charge to the total volume is expressed on the right-hand side of the equation. In the experiments, the motion of a finite liquid volume in the capillary is constrained by the liquid–gas interfaces at both ends. From a macroscopic perspective, the displacement length $L(r)$ remains independent of the capillary radius r and is equal to the liquid travel length, $L(r) = L_0$. This outcome is a direct consequence of the assumption that the charge density is decoupled from the flow velocity. The total charge within the finite volume is constant and entirely displaced along with the fluid. Therefore, such models are not directly applicable to describing the phenomena observed in our experiments. A more appropriate model for the present study may require a refined treatment of the charge adsorption at the interface or a more detailed characterization of the flow field.

SI Note S6: Charge Response in Sequential Pipetting for APTES-Modified Capillaries.

To examine the generality of the inter-cycle charge conservation behavior discussed in the main text, we performed analogous continuous uptake–release experiments using APTES (2 s)–ODTS and APTES (5 s)–ODTS modified capillaries.

Figures S8a and S8b show representative results obtained from 15 continuous measurements in which the uptake and release rates were varied. Similar to the ODTS case discussed in the main text (Figure 5a), changes in the release rate produced an immediate and pronounced response in the release charge Q_4 whereas the uptake charge Q_2 in the same cycle remained largely unchanged. Instead, the uptake charge in the subsequent cycle exhibited comparable magnitudes and opposite signs. This behavior is consistent with the approximate inter-cycle relation $Q_2^n \approx -Q_4^{n-1}$ introduced in the main text, indicating that the charge carried away during liquid release influences the subsequent uptake. While the absolute magnitudes of the charges differ between ODTS, APTES (2 s)–ODTS, and APTES (5 s)–ODTS capillaries, reflecting differences in surface chemistry, the qualitative inter-cycle coupling remains robust across all samples.

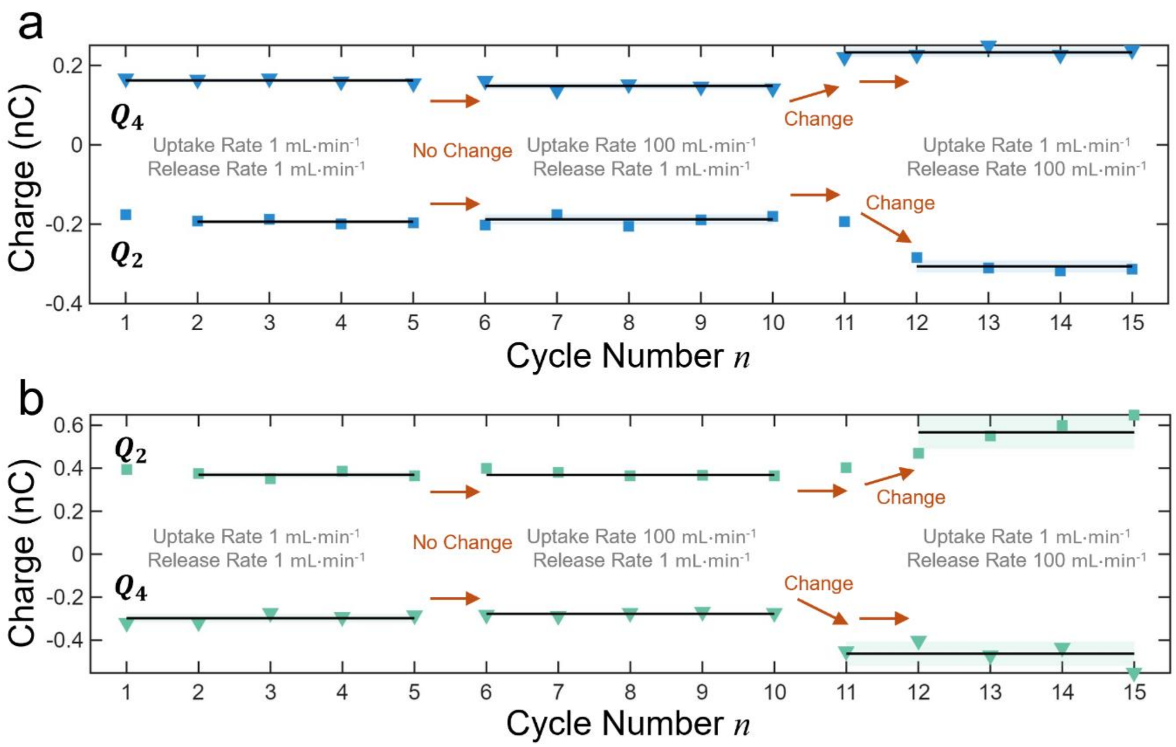

Figure S8. Inter cycle charge conservation in sequential pipetting. (**a**) Charges Q_2 (uptake) and Q_4 (release) recorded for 15 consecutive cycles for the APTES (2 s)-ODTS coated capillary. Three combinations of uptake/release rate adopted as indicated in the figure. The red arrows highlight the charge response to the rate change. Changes in the uptake rate have no effect on Q_2 and Q_4 (n = 6). When the release rate is increased from 1 to 100 mL·min^{-1} at cycle n = 11, showing that Q_4 rises immediately while Q_2 follows in the next cycle. Mean value (black line) and standard deviation (shaded area, not obvious due to a small value) relating to stable results were presented. Because there is no release history before uptake at n = 1, the first Q_2 value is different from the 2nd

to 5th, and was not included in calculating mean and standard deviation. **(b)** The same test for the capillary with APTES (5 s)-ODTS treatment.

Table S3. Reference values of $\Delta\theta_4$ associated with changes in the release

Release Rate at Cycle *n*-1 ($mL \cdot min^{-1}$)	Release Rate at Cycle *n* ($mL \cdot min^{-1}$)	ODTS	APTES (2 s)-ODTS	APTES (5 s)-ODTS
1	10	0.12 ± 0.03	0.03 ± 0.01	-0.11 ± 0.06
	100	0.18 ± 0.05	0.08 ± 0.02	-0.19 ± 0.06
10	1	-0.14 ± 0.02	-0.03 ± 0.01	0.15 ± 0.03
	100	0.08 ± 0.04	0.04 ± 0.02	-0.05 ± 0.03
100	1	-0.18 ± 0.05	-0.07 ± 0.02	0.17 ± 0.08
	10	-0.07 ± 0.04	-0.05 ± 0.02	0.04 ± 0.03

References

(1) Nauruzbayeva, J.; Sun, Z.; Gallo, A.; Ibrahim, M.; Santamarina, J. C.; Mishra, H. Electrification at water–hydrophobe interfaces. *Nature Communications* **2020**, *11* (1), 5285. DOI: 10.1038/s41467-020-19054-8.

(2) Lowell, J.; Akande, A. R. Contact electrification-why is it variable? *Journal of Physics D: Applied Physics* **1988**, *21* (1), 125-137. DOI: 10.1088/0022-3727/21/1/018.

(3) Lowell, J.; Truscott, W. S. Triboelectrification of Identical Insulators .1. An Experimental Investigation. *J Phys D Appl Phys* **1986**, *19* (7), 1273-1280. DOI: Doi 10.1088/0022-3727/19/7/017.

(4) Lowell, J.; Truscott, W. S. Triboelectrification of Identical Insulators .2. Theory and Further Experiments. *J Phys D Appl Phys* **1986**, *19* (7), 1281-1298. DOI: Doi 10.1088/0022-3727/19/7/018.

(5) Liu, C.; Bard, A. J. Electrostatic electrochemistry at insulators. *Nature Materials* **2008**, *7*, 505, Article. DOI: 10.1038/nmat2160

https://www.nature.com/articles/nmat2160#supplementary-information.

(6) Liu, C. Y.; Bard, A. J. Chemical Redox Reactions Induced by Cryptoelectrons on a PMMA Surface. *J Am Chem Soc* **2009**, *131* (18), 6397-6401. DOI: 10.1021/ja806785x.

(7) Schmid, M.; Rath, D.; Diebold, U. Why and how Savitzky–Golay filters should be replaced. *ACS Measurement Science Au* **2022**, *2* (2), 185-196.

(8) Berghaus, D.; Cannon, J. Obtaining derivatives from experimental data using smoothed-spline functions: The smoothed cubic spline is presented for use in obtaining high-quality derivatives from experimental data and example applications are shown for scattered-light photoelasticity and the bending of beams. *Experimental Mechanics* **1973**, *13* (1), 38-42.
